# P-2255. Peri-Transplant Antibiotic Use and Multidrug Resistant Infections in Liver Transplantation

**DOI:** 10.1093/ofid/ofae631.2408

**Published:** 2025-01-29

**Authors:** Caitlin A Contag, YaoWei Deng, Guillermo Rodriguez-Nava, Alex Zimmet, Sa Shen, Joanna K Nelson, Aruna Subramanian, Allison Kwong, Jessica Ferguson

**Affiliations:** Stanford University, Palo Alto, California; Stanford University, Palo Alto, California; Stanford University School of Medicine, Stanford, CA; Stanford Healthcare, Stanford University School of Medicine, Palo Alto, California; Quantitative Sciences Unit, Stanford, California; Stanford University, Palo Alto, California; Stanford University, Palo Alto, California; Stanford University, Palo Alto, California; Stanford University, Palo Alto, California

## Abstract

**Background:**

Understanding antibiotic use patterns and risk of infection with multi-drug resistant organisms (MDROs) in the peri-transplant period is crucial to successful post-liver transplant care.

**Figure:**
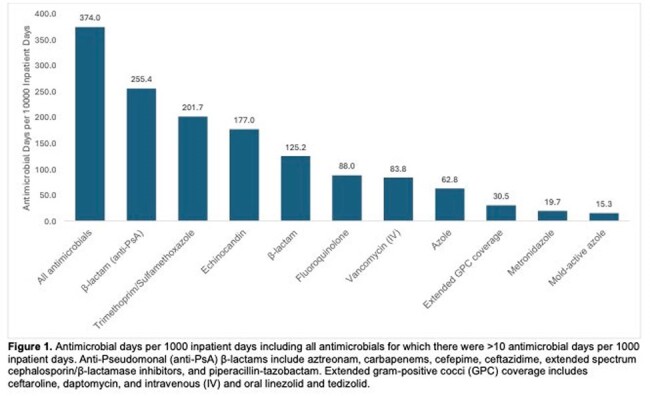

**Methods:**

Clinical data from patients who underwent liver transplant after the initiation of ICD-10 coding were obtained using Stanford University’s central database. Data were processed and analyzed in R studio and Excel.

**Figure:**
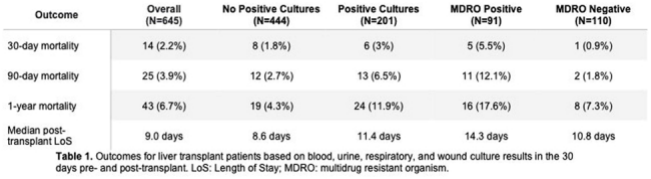

**Results:**

During the study period, 645 patients underwent liver transplantation. The median age at transplantation was 60.8 years (IQR 18.9, 82.4) and 222/645 (34%) were female. In the 30-day pre-transplant period, 21/645 (3%) patients developed bacteremia or fungemia, and in the 30-day post-transplant period, 30/645 (5%) patients developed bacteremia or fungemia. *Enterococcus faecium* was the most common cause of bacteremia (25/60 positive blood cultures), followed by enteric gram-negative rods (16/60 positive blood cultures). All *E. faecium* causing bacteremia was vancomycin resistant (25/25, 100%), and 9/16 (56%) gram negative rod isolates from blood were MDROs.

The overall antibiotic days per thousand inpatient days in the 30-day pre- and post-transplant period was 374. The most used group of antimicrobials was anti-Pseudomonal β-lactams, with 255.4 days of use per thousand inpatient days (Figure 1). Vancomycin had 83.8 days of use per thousand inpatient days but was more frequently used in patients who had an MDRO isolated from at least one body site than in patients who did not have an MDRO isolated (104.7 days vs 76.8 days per thousand inpatient days).

Patients with at least one MDRO isolated 30 days pre- or post-transplant had significantly higher 90-day and 1-year mortality compared with patients who had positive cultures but did not have an MDRO isolated (12.1% vs 1.8%, p=0.01; 17.6% vs 7.3%, p=0.02), as well as a longer length of post-transplant hospitalization that did not meet statistical significance (14.3 days vs 10.8 days, Table 1).

**Conclusion:**

Liver transplant patients are at high risk for colonization and infection with MDROs in the peri-transplant period and have high rates of antimicrobial use. For this patient cohort, presence of an MDRO is associated with higher rates of mortality and longer lengths of stay in the post-transplant period.

**Disclosures:**

All Authors: No reported disclosures

